# Bis(μ-2-phen­oxy­propionato-κ^2^ *O*:*O*′)bis­[(1,10-phenanthroline-κ^2^ *N*,*N*′)bis­(2-phen­oxy­propionato-κ^2^ *O*,*O*′)samarium(III)]

**DOI:** 10.1107/S1600536811034829

**Published:** 2011-08-31

**Authors:** Jin-Bei Shen, Jia-Lu Liu, Guo-Liang Zhao

**Affiliations:** a College of Chemistry and Life Sciences, Zhejiang Normal University, Jinhua 321004, Zhejiang, People’s Republic of China; b Zhejiang Normal University Xingzhi College, Jinhua, Zhejiang 321004, People’s Republic of China

## Abstract

The dimeric title compound, [Sm_2_(C_9_H_9_O_3_)_6_(C_12_H_8_N_2_)_2_], is centrosymmetric and is composed of six 2-phen­oxy­propionate anions and two 1,10-phenanthroline ligands. The Sm^III^ atom is coordinated by two O atoms from two bridging anions, four O atoms from two chelating anions and the N atoms of the *N*-heterocycle in a distorted dodeca­hedral geometry.

## Related literature

For the biological activity of phen­oxy­alkanoic acids, see: Markus & Buser (1997 ▶). For bond lengths and angles in related structures, see: Ye *et al.* (2010 ▶).
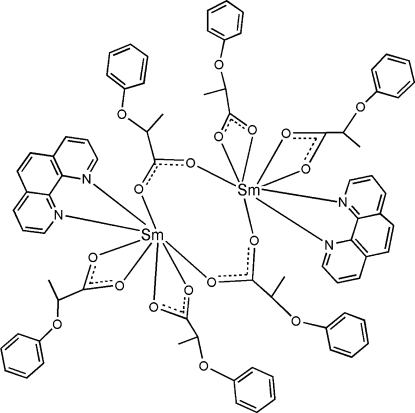


## Experimental

### 

#### Crystal data

[Sm_2_(C_9_H_9_O_3_)_6_(C_12_H_8_N_2_)_2_]
*M* *_r_* = 1652.08
Triclinic, 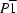

*a* = 11.3589 (6) Å
*b* = 12.2144 (6) Å
*c* = 14.1282 (8) Å
α = 99.111 (3)°
β = 91.116 (3)°
γ = 114.381 (3)°
*V* = 1754.98 (16) Å^3^
*Z* = 1
Mo *K*α radiation
μ = 1.73 mm^−1^
*T* = 296 K
0.40 × 0.29 × 0.05 mm

#### Data collection

Bruker APEXII area-detector diffractometer
Absorption correction: multi-scan (*SADABS*; Sheldrick, 1996 ▶) *T*_min_ = 0.556, *T*_max_ = 0.919
23059 measured reflections
6164 independent reflections
5585 reflections with *I* > 2σ(*I*)
*R*_int_ = 0.028

#### Refinement

*R*[*F* ^2^ > 2σ(*F* ^2^)] = 0.029
*wR*(*F* ^2^) = 0.079
*S* = 1.08
6164 reflections
460 parameters
18 restraints
H-atom parameters constrained
Δρ_max_ = 0.65 e Å^−3^
Δρ_min_ = −0.70 e Å^−3^

### 

Data collection: *APEX2* (Bruker, 2006 ▶); cell refinement: *SAINT* (Bruker, 2006 ▶); data reduction: *SAINT*; program(s) used to solve structure: *SHELXS97* (Sheldrick, 2008 ▶); program(s) used to refine structure: *SHELXL97* (Sheldrick, 2008 ▶); molecular graphics: *SHELXTL* (Sheldrick, 2008 ▶); software used to prepare material for publication: *SHELXL97*.

## Supplementary Material

Crystal structure: contains datablock(s) I, global. DOI: 10.1107/S1600536811034829/ng5218sup1.cif

Structure factors: contains datablock(s) I. DOI: 10.1107/S1600536811034829/ng5218Isup2.hkl

Additional supplementary materials:  crystallographic information; 3D view; checkCIF report

## Figures and Tables

**Fig. 1.**
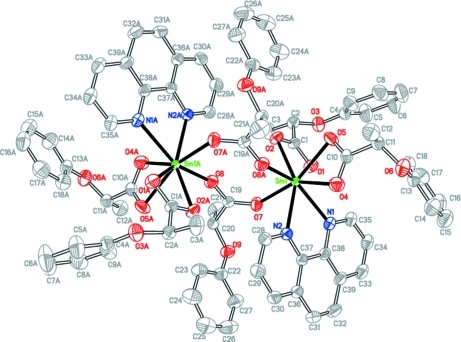
The molecular structure of the title complex, showing the atom-labelling scheme. Displacement ellipsoids are drawn at the 30% probability level.

**Fig. 2.**
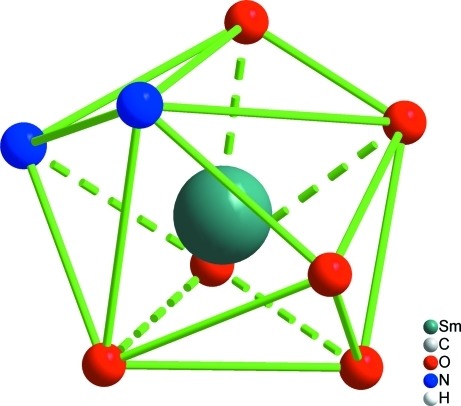
The coordination environment of the Sm(III).

## supplementary crystallographic information

### Comment

The group of phenoxyalkanoic acids includes a considerable number of important herbicides. The desired biological activity is largely dependent on the length of the carbon chain of the alkanoic acid, the nature of the phenoxy group, and the position of its attachment to the carbon chain (Markus *et al.*, 1997). The structures of 2-phenoxypropionic acid complexes coupled with their special functionality are our interests. Here, we describe a new Sm^III^ complex.

The dimeric title compound [Sm_2_(C_9_H_9_O_3_)_6_(C_12_H_8_N_2_)_2_] (Scheme I) is centrosymmetric and it comprises of six 2-phenoxypropionate anions and two 1,10-phenanthroline ligands. The Sm^III^ atoms are bridged by two anions. The Sm^III^ atom coordinated by two O atoms from two bridging anions, four O atoms from two chelating anions and the N atoms of the *N*-heterocycle in a dodecahedronal geometry (Fig. 1).

The Sm—Sm separation is 5.1452 (3) Å.

The analysis of structural features indicates that the Sm ion adopts a distorted dodecahedron geometry (Fig. 2) (Ye *et al.*, 2010). Such a geometry is seldom reported and is interesting in lanthanide carboxylate complexes. The *L* ligands are coordinated to the Sm^III^ ions in two different modes: chelating and bridging. The Sm—O distances are all within the range 2.199 (3)–2.396 (3) Å, and the Sm—N distances range from 2.459 (3)–2.480 (3) Å, all of which are within the range of those of other eight-coordinated Sm^III^ complexes with carboxylic donor ligands and 1,10-phenanthroline (Ye *et al.*, 2010). The most significant intermolecular interactions are C—H···O hydrogen bonds(Table 2) and weak π···π aromatic interactions from the phenananthroline molecules and aromatic rings of the anionic ligands.

### Experimental

Reagents and solvents used were of commercially available quality. 2-Phenoxypropionic acid (1.5 mmol), Sm(NO_3_)_3_^.^6H_2_O (0.5 mmol) and 1,10-phenanthroline (0.5 mmol) were dissolved in 20 ml e nthanol; 10 ml water was added to the solution. The solution was stirred for 12 h at room temperature. Colorless crystals were obtained after several days.

### Refinement

The H atoms bonded to C and N atoms were positioned geometrically and refined using a riding model [aliphatic C—H =0.96 Å (*U*_iso_(H) = 1.5*U*_eq_(C)), aromatic C—H = 0.93 Å (*U*_iso_(H) = 1.2*U*_eq_(C))].

### Crystal data

**Table:**

[Sm_2_(C_9_H_9_O_3_)_6_(C_12_H_8_N_2_)_2_]	*Z* = 1
*M**_r_* = 1652.08	*F*(000) = 834
Triclinic, *P*1	*D*_x_ = 1.563 Mg m^−^^3^
Hall symbol: -P 1	Mo *K*α radiation, λ = 0.71073 Å
*a* = 11.3589 (6) Å	Cell parameters from 9923 reflections
*b* = 12.2144 (6) Å	θ = 1.9–25.0°
*c* = 14.1282 (8) Å	µ = 1.73 mm^−^^1^
α = 99.111 (3)°	*T* = 296 K
β = 91.116 (3)°	Block, colourless
γ = 114.381 (3)°	0.40 × 0.29 × 0.05 mm
*V* = 1754.98 (16) Å^3^	

### Data collection

**Table:**

Bruker APEXII area-detector diffractometer	6164 independent reflections
Radiation source: fine-focus sealed tube	5585 reflections with *I* > 2σ(*I*)
graphite	*R*_int_ = 0.028
φ and ω scans	θ_max_ = 25.0°, θ_min_ = 1.9°
Absorption correction: multi-scan (*SADABS*; Sheldrick, 1996)	*h* = −13→13
*T*_min_ = 0.556, *T*_max_ = 0.919	*k* = −14→14
23059 measured reflections	*l* = −16→16

### Refinement

**Table:**

Refinement on *F*^2^	Primary atom site location: structure-invariant direct methods
Least-squares matrix: full	Secondary atom site location: difference Fourier map
*R*[*F*^2^ > 2σ(*F*^2^)] = 0.029	Hydrogen site location: inferred from neighbouring sites
*wR*(*F*^2^) = 0.079	H-atom parameters constrained
*S* = 1.08	*w* = 1/[σ^2^(*F*_o_^2^) + (0.0504*P*)^2^ + 0.4271*P*] where *P* = (*F*_o_^2^ + 2*F*_c_^2^)/3
6164 reflections	(Δ/σ)_max_ = 0.002
460 parameters	Δρ_max_ = 0.65 e Å^−^^3^
18 restraints	Δρ_min_ = −0.70 e Å^−^^3^

### Special details

**Table:**

Geometry. All e.s.d.'s (except the e.s.d. in the dihedral angle between two l.s. planes) are estimated using the full covariance matrix. The cell e.s.d.'s are taken into account individually in the estimation of e.s.d.'s in distances, angles and torsion angles; correlations between e.s.d.'s in cell parameters are only used when they are defined by crystal symmetry. An approximate (isotropic) treatment of cell e.s.d.'s is used for estimating e.s.d.'s involving l.s. planes.
Refinement. Refinement of *F*^2^ against ALL reflections. The weighted *R*-factor *wR* and goodness of fit *S* are based on *F*^2^, conventional *R*-factors *R* are based on *F*, with *F* set to zero for negative *F*^2^. The threshold expression of *F*^2^ > σ(*F*^2^) is used only for calculating *R*-factors(gt) *etc*. and is not relevant to the choice of reflections for refinement. *R*-factors based on *F*^2^ are statistically about twice as large as those based on *F*, and *R*- factors based on ALL data will be even larger.

### Fractional atomic coordinates and isotropic or equivalent isotropic displacement parameters (Å^2^)

**Table:**

	*x*	*y*	*z*	*U*_iso_*/*U*_eq_	
Sm1	0.821883 (15)	0.887562 (13)	0.604388 (11)	0.03012 (8)	
C19	0.9324 (4)	0.8368 (3)	0.3955 (3)	0.0417 (9)	
O9	0.7694 (3)	0.6931 (3)	0.2678 (2)	0.0622 (8)	
N2	0.6794 (3)	0.9250 (3)	0.4929 (2)	0.0459 (8)	
O2	1.0181 (3)	0.8723 (2)	0.6407 (2)	0.0521 (7)	
O1	0.8364 (3)	0.7135 (2)	0.6450 (2)	0.0582 (7)	
O8	1.0419 (3)	0.9270 (2)	0.4104 (2)	0.0568 (7)	
N1	0.5970 (3)	0.7250 (3)	0.5784 (3)	0.0507 (8)	
O3	0.9648 (3)	0.6020 (3)	0.7353 (2)	0.0625 (8)	
C1	0.9580 (4)	0.7664 (3)	0.6573 (3)	0.0444 (8)	
O6	0.6034 (3)	0.9804 (3)	0.8792 (2)	0.0716 (9)	
C37	0.5571 (4)	0.8368 (4)	0.4654 (3)	0.0470 (9)	
C20	0.9041 (4)	0.7522 (4)	0.2987 (3)	0.0506 (10)	
H20A	0.9517	0.7981	0.2503	0.061*	
O5	0.8677 (3)	0.9529 (3)	0.7753 (2)	0.0679 (9)	
O7	0.8510 (3)	0.8087 (3)	0.4545 (2)	0.0557 (7)	
O4	0.7112 (3)	0.9757 (3)	0.7047 (2)	0.0663 (8)	
C10	0.7742 (4)	0.9820 (4)	0.7803 (3)	0.0492 (8)	
C2	1.0366 (4)	0.7021 (4)	0.6887 (3)	0.0534 (10)	
H2A	1.1122	0.7612	0.7320	0.064*	
C11	0.7397 (4)	1.0285 (4)	0.8767 (3)	0.0573 (11)	
H11A	0.7780	1.0048	0.9282	0.069*	
C38	0.5137 (4)	0.7313 (4)	0.5106 (3)	0.0492 (10)	
C22	0.7137 (4)	0.7577 (4)	0.2246 (3)	0.0554 (11)	
C39	0.3889 (4)	0.6380 (4)	0.4844 (3)	0.0594 (11)	
C35	0.5525 (5)	0.6292 (4)	0.6230 (3)	0.0589 (11)	
H35A	0.6078	0.6249	0.6702	0.071*	
C30	0.5161 (5)	0.9533 (5)	0.3591 (3)	0.0643 (12)	
H30A	0.4620	0.9639	0.3147	0.077*	
C28	0.7173 (4)	1.0236 (4)	0.4537 (3)	0.0553 (10)	
H28A	0.8009	1.0841	0.4718	0.066*	
C13	0.5368 (5)	0.8547 (4)	0.8707 (3)	0.0605 (11)	
C33	0.3482 (5)	0.5388 (4)	0.5327 (4)	0.0666 (13)	
H33A	0.2660	0.4750	0.5167	0.080*	
C36	0.4727 (4)	0.8471 (4)	0.3979 (3)	0.0561 (11)	
C3	1.0824 (6)	0.6470 (5)	0.6017 (4)	0.0744 (14)	
H3A	1.1328	0.6069	0.6222	0.112*	
H3B	1.1348	0.7105	0.5685	0.112*	
H3C	1.0086	0.5886	0.5593	0.112*	
C29	0.6393 (5)	1.0424 (5)	0.3868 (4)	0.0681 (13)	
H29A	0.6701	1.1138	0.3614	0.082*	
C34	0.4293 (5)	0.5363 (4)	0.6030 (4)	0.0653 (12)	
H34A	0.4022	0.4725	0.6374	0.078*	
C4	0.9277 (5)	0.6275 (4)	0.8253 (3)	0.0579 (11)	
C23	0.7806 (5)	0.8736 (4)	0.2077 (4)	0.0692 (13)	
H23A	0.8681	0.9173	0.2294	0.083*	
C9	0.9416 (6)	0.7410 (5)	0.8705 (4)	0.0743 (14)	
H9A	0.9800	0.8088	0.8413	0.089*	
C12	0.7871 (6)	1.1663 (4)	0.8946 (4)	0.0785 (15)	
H12A	0.7644	1.1931	0.9563	0.118*	
H12B	0.7471	1.1890	0.8453	0.118*	
H12C	0.8797	1.2040	0.8935	0.118*	
C21	0.9466 (6)	0.6517 (5)	0.3091 (4)	0.0810 (16)	
H21A	0.9296	0.5975	0.2483	0.122*	
H21B	0.8993	0.6068	0.3563	0.122*	
H21C	1.0379	0.6874	0.3292	0.122*	
C18	0.5918 (6)	0.7774 (5)	0.8792 (4)	0.0735 (14)	
H18A	0.6817	0.8066	0.8883	0.088*	
C32	0.3066 (5)	0.6504 (5)	0.4112 (4)	0.0773 (15)	
H32A	0.2243	0.5880	0.3918	0.093*	
C31	0.3475 (5)	0.7507 (5)	0.3710 (4)	0.0744 (14)	
H31A	0.2926	0.7570	0.3246	0.089*	
C27	0.5839 (5)	0.6938 (5)	0.1956 (4)	0.0761 (14)	
H27A	0.5379	0.6161	0.2093	0.091*	
C17	0.5140 (7)	0.6548 (6)	0.8743 (4)	0.0881 (17)	
H17A	0.5521	0.6020	0.8821	0.106*	
C25	0.5894 (7)	0.8629 (6)	0.1260 (4)	0.0911 (18)	
H25A	0.5479	0.8978	0.0915	0.109*	
C24	0.7171 (7)	0.9254 (6)	0.1581 (4)	0.0879 (18)	
H24A	0.7626	1.0043	0.1464	0.105*	
C5	0.8736 (7)	0.5269 (6)	0.8683 (5)	0.099 (2)	
H5A	0.8649	0.4509	0.8362	0.118*	
C16	0.3792 (7)	0.6083 (5)	0.8578 (4)	0.0905 (18)	
H16A	0.3267	0.5255	0.8539	0.109*	
C8	0.8972 (6)	0.7520 (6)	0.9608 (4)	0.0894 (17)	
H8A	0.9042	0.8276	0.9924	0.107*	
C14	0.4038 (6)	0.8110 (5)	0.8534 (4)	0.0838 (16)	
H14A	0.3668	0.8646	0.8457	0.101*	
C26	0.5222 (6)	0.7471 (6)	0.1455 (4)	0.0929 (19)	
H26A	0.4344	0.7042	0.1248	0.112*	
C15	0.3266 (6)	0.6900 (6)	0.8476 (5)	0.0959 (19)	
H15A	0.2371	0.6618	0.8366	0.115*	
C6	0.8321 (9)	0.5382 (7)	0.9594 (5)	0.126 (3)	
H6A	0.7979	0.4709	0.9897	0.151*	
C7	0.8421 (7)	0.6498 (7)	1.0040 (5)	0.113 (2)	
H7A	0.8116	0.6575	1.0642	0.136*	

### Atomic displacement parameters (Å^2^)

**Table:**

	*U*^11^	*U*^22^	*U*^33^	*U*^12^	*U*^13^	*U*^23^
Sm1	0.02637 (11)	0.03024 (10)	0.03149 (12)	0.00984 (8)	0.00305 (7)	0.00517 (7)
C19	0.041 (2)	0.0375 (18)	0.048 (2)	0.0173 (17)	0.0013 (18)	0.0091 (16)
O9	0.0541 (19)	0.0538 (16)	0.066 (2)	0.0129 (14)	−0.0041 (15)	0.0035 (14)
N2	0.0414 (19)	0.0523 (18)	0.0432 (19)	0.0195 (15)	0.0042 (14)	0.0075 (14)
O2	0.0368 (12)	0.0486 (15)	0.074 (2)	0.0189 (12)	0.0051 (13)	0.0172 (13)
O1	0.0499 (18)	0.0466 (14)	0.080 (2)	0.0178 (13)	−0.0007 (15)	0.0243 (13)
O8	0.0521 (18)	0.0442 (15)	0.0641 (19)	0.0106 (14)	−0.0015 (14)	0.0101 (13)
N1	0.0422 (19)	0.0498 (18)	0.058 (2)	0.0162 (15)	0.0107 (16)	0.0113 (16)
O3	0.075 (2)	0.0549 (16)	0.066 (2)	0.0332 (16)	0.0171 (16)	0.0196 (14)
C1	0.040 (2)	0.045 (2)	0.052 (2)	0.0203 (16)	0.0015 (17)	0.0091 (16)
O6	0.066 (2)	0.070 (2)	0.083 (2)	0.0318 (17)	0.0235 (18)	0.0153 (17)
C37	0.039 (2)	0.059 (2)	0.042 (2)	0.0212 (19)	0.0057 (17)	0.0032 (18)
C20	0.044 (2)	0.050 (2)	0.052 (2)	0.0164 (19)	0.0068 (19)	0.0039 (18)
O5	0.079 (2)	0.103 (2)	0.0377 (10)	0.058 (2)	0.0036 (14)	0.0052 (14)
O7	0.0533 (18)	0.0556 (16)	0.0523 (18)	0.0178 (14)	0.0152 (15)	0.0069 (13)
O4	0.0654 (11)	0.0731 (11)	0.0633 (11)	0.0335 (9)	0.0019 (8)	0.0092 (9)
C10	0.055 (2)	0.051 (2)	0.0451 (13)	0.0270 (19)	0.0130 (14)	0.0042 (16)
C2	0.048 (2)	0.050 (2)	0.064 (3)	0.0214 (19)	0.006 (2)	0.0150 (19)
C11	0.058 (3)	0.065 (3)	0.050 (3)	0.029 (2)	0.007 (2)	0.006 (2)
C38	0.040 (2)	0.056 (2)	0.048 (2)	0.0190 (19)	0.0083 (18)	0.0011 (18)
C22	0.059 (3)	0.056 (2)	0.048 (2)	0.027 (2)	−0.001 (2)	−0.0026 (19)
C39	0.041 (2)	0.062 (3)	0.062 (3)	0.013 (2)	0.007 (2)	0.000 (2)
C35	0.055 (3)	0.057 (2)	0.063 (3)	0.019 (2)	0.016 (2)	0.016 (2)
C30	0.055 (3)	0.092 (3)	0.056 (3)	0.041 (3)	0.004 (2)	0.015 (2)
C28	0.052 (3)	0.059 (2)	0.057 (3)	0.023 (2)	0.005 (2)	0.018 (2)
C13	0.063 (3)	0.057 (3)	0.060 (3)	0.023 (2)	0.020 (2)	0.009 (2)
C33	0.048 (3)	0.058 (3)	0.080 (4)	0.010 (2)	0.015 (2)	0.005 (2)
C36	0.044 (2)	0.074 (3)	0.051 (3)	0.027 (2)	0.0055 (19)	0.007 (2)
C3	0.086 (4)	0.072 (3)	0.085 (4)	0.047 (3)	0.036 (3)	0.025 (3)
C29	0.066 (3)	0.080 (3)	0.069 (3)	0.036 (3)	0.008 (3)	0.026 (3)
C34	0.057 (3)	0.053 (2)	0.078 (3)	0.014 (2)	0.020 (3)	0.014 (2)
C4	0.060 (3)	0.069 (3)	0.052 (3)	0.032 (2)	0.010 (2)	0.016 (2)
C23	0.071 (3)	0.064 (3)	0.069 (3)	0.029 (3)	−0.006 (3)	0.003 (2)
C9	0.092 (4)	0.082 (3)	0.062 (3)	0.049 (3)	0.007 (3)	0.014 (3)
C12	0.086 (4)	0.064 (3)	0.074 (4)	0.028 (3)	0.008 (3)	−0.008 (2)
C21	0.083 (4)	0.065 (3)	0.102 (4)	0.044 (3)	0.011 (3)	−0.003 (3)
C18	0.075 (3)	0.070 (3)	0.078 (4)	0.032 (3)	0.011 (3)	0.015 (3)
C32	0.042 (3)	0.090 (4)	0.080 (4)	0.013 (3)	−0.002 (2)	0.003 (3)
C31	0.049 (3)	0.105 (4)	0.064 (3)	0.028 (3)	−0.007 (2)	0.014 (3)
C27	0.060 (3)	0.077 (3)	0.080 (4)	0.026 (3)	−0.002 (3)	−0.004 (3)
C17	0.107 (5)	0.083 (4)	0.085 (4)	0.049 (4)	0.007 (4)	0.022 (3)
C25	0.111 (5)	0.097 (4)	0.079 (4)	0.066 (4)	−0.009 (4)	−0.006 (3)
C24	0.104 (5)	0.079 (4)	0.087 (4)	0.048 (4)	−0.010 (4)	0.006 (3)
C5	0.123 (5)	0.082 (4)	0.085 (4)	0.032 (4)	0.036 (4)	0.028 (3)
C16	0.095 (5)	0.072 (3)	0.090 (4)	0.018 (3)	0.033 (3)	0.018 (3)
C8	0.101 (5)	0.109 (5)	0.069 (4)	0.059 (4)	0.007 (3)	0.004 (3)
C14	0.078 (4)	0.080 (4)	0.096 (4)	0.036 (3)	0.013 (3)	0.016 (3)
C26	0.078 (4)	0.116 (5)	0.086 (4)	0.055 (4)	−0.015 (3)	−0.015 (4)
C15	0.075 (4)	0.093 (4)	0.107 (5)	0.022 (3)	0.020 (3)	0.017 (4)
C6	0.169 (8)	0.105 (5)	0.090 (5)	0.036 (5)	0.063 (5)	0.032 (4)
C7	0.124 (6)	0.134 (6)	0.071 (4)	0.042 (5)	0.035 (4)	0.019 (4)

### Geometric parameters (Å, °)

**Table:**

Sm1—O8^i^	2.199 (3)	C28—H28A	0.9300
Sm1—O7	2.272 (3)	C13—C18	1.347 (7)
Sm1—O4	2.335 (3)	C13—C14	1.380 (7)
Sm1—O1	2.356 (3)	C33—C34	1.355 (7)
Sm1—O2	2.366 (3)	C33—H33A	0.9300
Sm1—O5	2.396 (3)	C36—C31	1.417 (7)
Sm1—N2	2.459 (3)	C3—H3A	0.9600
Sm1—N1	2.480 (3)	C3—H3B	0.9600
Sm1—C1	2.708 (4)	C3—H3C	0.9600
Sm1—C10	2.731 (4)	C29—H29A	0.9300
C19—O7	1.237 (5)	C34—H34A	0.9300
C19—O8	1.261 (5)	C4—C9	1.374 (6)
C19—C20	1.517 (5)	C4—C5	1.373 (7)
O9—C22	1.395 (5)	C23—C24	1.382 (7)
O9—C20	1.420 (5)	C23—H23A	0.9300
N2—C28	1.318 (5)	C9—C8	1.389 (7)
N2—C37	1.361 (5)	C9—H9A	0.9300
O2—C1	1.251 (5)	C12—H12A	0.9600
O1—C1	1.255 (5)	C12—H12B	0.9600
O8—Sm1^i^	2.199 (3)	C12—H12C	0.9600
N1—C35	1.333 (5)	C21—H21A	0.9600
N1—C38	1.366 (5)	C21—H21B	0.9600
O3—C4	1.376 (5)	C21—H21C	0.9600
O3—C2	1.430 (5)	C18—C17	1.379 (8)
C1—C2	1.512 (5)	C18—H18A	0.9300
O6—C13	1.387 (5)	C32—C31	1.342 (8)
O6—C11	1.415 (5)	C32—H32A	0.9300
C37—C36	1.396 (6)	C31—H31A	0.9300
C37—C38	1.436 (6)	C27—C26	1.384 (8)
C20—C21	1.517 (6)	C27—H27A	0.9300
C20—H20A	0.9800	C17—C16	1.396 (9)
O5—C10	1.252 (5)	C17—H17A	0.9300
O4—C10	1.247 (5)	C25—C24	1.357 (8)
C10—C11	1.513 (6)	C25—C26	1.378 (9)
C2—C3	1.512 (6)	C25—H25A	0.9300
C2—H2A	0.9800	C24—H24A	0.9300
C11—C12	1.516 (6)	C5—C6	1.385 (9)
C11—H11A	0.9800	C5—H5A	0.9300
C38—C39	1.400 (6)	C16—C15	1.381 (8)
C22—C23	1.365 (7)	C16—H16A	0.9300
C22—C27	1.370 (6)	C8—C7	1.390 (9)
C39—C33	1.400 (7)	C8—H8A	0.9300
C39—C32	1.447 (7)	C14—C15	1.359 (8)
C35—C34	1.377 (6)	C14—H14A	0.9300
C35—H35A	0.9300	C26—H26A	0.9300
C30—C29	1.372 (7)	C15—H15A	0.9300
C30—C36	1.390 (7)	C6—C7	1.365 (9)
C30—H30A	0.9300	C6—H6A	0.9300
C28—C29	1.391 (6)	C7—H7A	0.9300
			
O8^i^—Sm1—O7	91.47 (11)	C27—C22—O9	114.6 (4)
O8^i^—Sm1—O4	88.17 (12)	C33—C39—C38	118.1 (4)
O7—Sm1—O4	149.77 (12)	C33—C39—C32	123.1 (4)
O8^i^—Sm1—O1	136.67 (11)	C38—C39—C32	118.7 (4)
O7—Sm1—O1	83.32 (11)	N1—C35—C34	123.8 (5)
O4—Sm1—O1	116.71 (11)	N1—C35—H35A	118.1
O8^i^—Sm1—O2	81.62 (10)	C34—C35—H35A	118.1
O7—Sm1—O2	80.95 (11)	C29—C30—C36	119.6 (4)
O4—Sm1—O2	128.74 (11)	C29—C30—H30A	120.2
O1—Sm1—O2	55.06 (10)	C36—C30—H30A	120.2
O8^i^—Sm1—O5	87.81 (12)	N2—C28—C29	123.8 (4)
O7—Sm1—O5	155.91 (12)	N2—C28—H28A	118.1
O4—Sm1—O5	54.29 (11)	C29—C28—H28A	118.1
O1—Sm1—O5	80.78 (11)	C18—C13—C14	120.3 (5)
O2—Sm1—O5	75.13 (10)	C18—C13—O6	125.2 (5)
O8^i^—Sm1—N2	82.55 (11)	C14—C13—O6	114.4 (4)
O7—Sm1—N2	74.33 (11)	C34—C33—C39	119.6 (4)
O4—Sm1—N2	75.66 (11)	C34—C33—H33A	120.2
O1—Sm1—N2	135.64 (10)	C39—C33—H33A	120.2
O2—Sm1—N2	150.16 (11)	C30—C36—C37	117.8 (4)
O5—Sm1—N2	129.29 (10)	C30—C36—C31	122.4 (4)
O8^i^—Sm1—N1	148.53 (12)	C37—C36—C31	119.7 (4)
O7—Sm1—N1	87.20 (11)	C2—C3—H3A	109.5
O4—Sm1—N1	77.76 (11)	C2—C3—H3B	109.5
O1—Sm1—N1	74.40 (11)	H3A—C3—H3B	109.5
O2—Sm1—N1	128.97 (10)	C2—C3—H3C	109.5
O5—Sm1—N1	105.65 (12)	H3A—C3—H3C	109.5
N2—Sm1—N1	66.81 (11)	H3B—C3—H3C	109.5
O8^i^—Sm1—C1	109.08 (11)	C30—C29—C28	118.6 (5)
O7—Sm1—C1	82.08 (12)	C30—C29—H29A	120.7
O4—Sm1—C1	126.40 (12)	C28—C29—H29A	120.7
O1—Sm1—C1	27.59 (10)	C33—C34—C35	119.2 (5)
O2—Sm1—C1	27.50 (10)	C33—C34—H34A	120.4
O5—Sm1—C1	75.45 (11)	C35—C34—H34A	120.4
N2—Sm1—C1	154.05 (11)	C9—C4—C5	121.4 (5)
N1—Sm1—C1	101.87 (11)	C9—C4—O3	125.2 (4)
O8^i^—Sm1—C10	89.10 (12)	C5—C4—O3	113.4 (4)
O7—Sm1—C10	176.79 (12)	C22—C23—C24	119.4 (5)
O4—Sm1—C10	27.08 (11)	C22—C23—H23A	120.3
O1—Sm1—C10	98.42 (12)	C24—C23—H23A	120.3
O2—Sm1—C10	102.26 (12)	C4—C9—C8	118.5 (5)
O5—Sm1—C10	27.27 (11)	C4—C9—H9A	120.7
N2—Sm1—C10	102.62 (12)	C8—C9—H9A	120.7
N1—Sm1—C10	90.66 (12)	C11—C12—H12A	109.5
C1—Sm1—C10	100.72 (12)	C11—C12—H12B	109.5
O7—C19—O8	125.5 (4)	H12A—C12—H12B	109.5
O7—C19—C20	118.2 (3)	C11—C12—H12C	109.5
O8—C19—C20	116.2 (4)	H12A—C12—H12C	109.5
C22—O9—C20	118.2 (3)	H12B—C12—H12C	109.5
C28—N2—C37	117.5 (4)	C20—C21—H21A	109.5
C28—N2—Sm1	123.5 (3)	C20—C21—H21B	109.5
C37—N2—Sm1	118.9 (3)	H21A—C21—H21B	109.5
C1—O2—Sm1	91.7 (2)	C20—C21—H21C	109.5
C1—O1—Sm1	92.0 (2)	H21A—C21—H21C	109.5
C19—O8—Sm1^i^	152.9 (3)	H21B—C21—H21C	109.5
C35—N1—C38	117.4 (4)	C13—C18—C17	119.6 (6)
C35—N1—Sm1	124.9 (3)	C13—C18—H18A	120.2
C38—N1—Sm1	117.6 (3)	C17—C18—H18A	120.2
C4—O3—C2	118.2 (3)	C31—C32—C39	120.8 (5)
O2—C1—O1	121.1 (3)	C31—C32—H32A	119.6
O2—C1—C2	118.0 (3)	C39—C32—H32A	119.6
O1—C1—C2	120.8 (3)	C32—C31—C36	121.3 (5)
O2—C1—Sm1	60.83 (19)	C32—C31—H31A	119.3
O1—C1—Sm1	60.40 (19)	C36—C31—H31A	119.3
C2—C1—Sm1	178.4 (3)	C22—C27—C26	118.8 (6)
C13—O6—C11	117.2 (4)	C22—C27—H27A	120.6
N2—C37—C36	122.7 (4)	C26—C27—H27A	120.6
N2—C37—C38	117.7 (4)	C18—C17—C16	121.2 (6)
C36—C37—C38	119.6 (4)	C18—C17—H17A	119.4
O9—C20—C21	106.4 (4)	C16—C17—H17A	119.4
O9—C20—C19	112.2 (3)	C24—C25—C26	119.0 (6)
C21—C20—C19	108.5 (4)	C24—C25—H25A	120.5
O9—C20—H20A	109.9	C26—C25—H25A	120.5
C21—C20—H20A	109.9	C25—C24—C23	121.0 (6)
C19—C20—H20A	109.9	C25—C24—H24A	119.5
C10—O5—Sm1	91.5 (2)	C23—C24—H24A	119.5
C19—O7—Sm1	138.2 (3)	C4—C5—C6	120.1 (6)
C10—O4—Sm1	94.4 (3)	C4—C5—H5A	119.9
O4—C10—O5	119.5 (4)	C6—C5—H5A	119.9
O4—C10—C11	119.8 (4)	C15—C16—C17	117.5 (6)
O5—C10—C11	120.6 (4)	C15—C16—H16A	121.3
O4—C10—Sm1	58.5 (2)	C17—C16—H16A	121.3
O5—C10—Sm1	61.3 (2)	C9—C8—C7	119.9 (6)
C11—C10—Sm1	176.6 (3)	C9—C8—H8A	120.1
O3—C2—C1	113.8 (3)	C7—C8—H8A	120.1
O3—C2—C3	105.3 (3)	C15—C14—C13	120.4 (6)
C1—C2—C3	109.8 (4)	C15—C14—H14A	119.8
O3—C2—H2A	109.3	C13—C14—H14A	119.8
C1—C2—H2A	109.3	C25—C26—C27	120.9 (6)
C3—C2—H2A	109.3	C25—C26—H26A	119.6
O6—C11—C10	110.6 (4)	C27—C26—H26A	119.6
O6—C11—C12	105.8 (4)	C14—C15—C16	121.0 (6)
C10—C11—C12	111.4 (4)	C14—C15—H15A	119.5
O6—C11—H11A	109.6	C16—C15—H15A	119.5
C10—C11—H11A	109.6	C7—C6—C5	119.0 (7)
C12—C11—H11A	109.6	C7—C6—H6A	120.5
N1—C38—C39	121.8 (4)	C5—C6—H6A	120.5
N1—C38—C37	118.4 (3)	C6—C7—C8	121.0 (6)
C39—C38—C37	119.8 (4)	C6—C7—H7A	119.5
C23—C22—C27	120.9 (5)	C8—C7—H7A	119.5
C23—C22—O9	124.5 (4)		
			
O8^i^—Sm1—N2—C28	5.0 (3)	O8^i^—Sm1—O4—C10	−91.6 (3)
O7—Sm1—N2—C28	−88.7 (3)	O7—Sm1—O4—C10	178.6 (2)
O4—Sm1—N2—C28	95.0 (3)	O1—Sm1—O4—C10	51.4 (3)
O1—Sm1—N2—C28	−151.3 (3)	O2—Sm1—O4—C10	−13.9 (3)
O2—Sm1—N2—C28	−53.5 (4)	O5—Sm1—O4—C10	−3.0 (2)
O5—Sm1—N2—C28	86.0 (3)	N2—Sm1—O4—C10	−174.4 (3)
N1—Sm1—N2—C28	177.6 (3)	N1—Sm1—O4—C10	116.8 (3)
C1—Sm1—N2—C28	−114.0 (4)	C1—Sm1—O4—C10	20.9 (3)
C10—Sm1—N2—C28	92.4 (3)	Sm1—O4—C10—O5	5.3 (4)
O8^i^—Sm1—N2—C37	−178.9 (3)	Sm1—O4—C10—C11	−176.6 (3)
O7—Sm1—N2—C37	87.4 (3)	Sm1—O5—C10—O4	−5.2 (4)
O4—Sm1—N2—C37	−88.9 (3)	Sm1—O5—C10—C11	176.8 (4)
O1—Sm1—N2—C37	24.8 (3)	O8^i^—Sm1—C10—O4	87.8 (3)
O2—Sm1—N2—C37	122.6 (3)	O1—Sm1—C10—O4	−135.1 (3)
O5—Sm1—N2—C37	−97.9 (3)	O2—Sm1—C10—O4	169.0 (2)
N1—Sm1—N2—C37	−6.3 (3)	O5—Sm1—C10—O4	174.7 (4)
C1—Sm1—N2—C37	62.1 (4)	N2—Sm1—C10—O4	5.6 (3)
C10—Sm1—N2—C37	−91.5 (3)	N1—Sm1—C10—O4	−60.8 (3)
O8^i^—Sm1—O2—C1	176.9 (2)	C1—Sm1—C10—O4	−163.0 (3)
O7—Sm1—O2—C1	−90.2 (2)	O8^i^—Sm1—C10—O5	−87.0 (3)
O4—Sm1—O2—C1	96.1 (3)	O4—Sm1—C10—O5	−174.7 (4)
O1—Sm1—O2—C1	−2.1 (2)	O1—Sm1—C10—O5	50.2 (3)
O5—Sm1—O2—C1	87.0 (2)	O2—Sm1—C10—O5	−5.8 (3)
N2—Sm1—O2—C1	−124.4 (3)	N2—Sm1—C10—O5	−169.1 (3)
N1—Sm1—O2—C1	−11.3 (3)	N1—Sm1—C10—O5	124.5 (3)
C10—Sm1—O2—C1	89.7 (2)	C1—Sm1—C10—O5	22.3 (3)
O8^i^—Sm1—O1—C1	0.6 (3)	C4—O3—C2—C1	−69.5 (5)
O7—Sm1—O1—C1	85.7 (3)	C4—O3—C2—C3	170.2 (4)
O4—Sm1—O1—C1	−118.1 (2)	O2—C1—C2—O3	160.7 (4)
O2—Sm1—O1—C1	2.1 (2)	O1—C1—C2—O3	−21.7 (6)
O5—Sm1—O1—C1	−76.2 (3)	O2—C1—C2—C3	−81.6 (5)
N2—Sm1—O1—C1	145.1 (2)	O1—C1—C2—C3	96.0 (5)
N1—Sm1—O1—C1	174.6 (3)	C13—O6—C11—C10	63.1 (5)
C10—Sm1—O1—C1	−97.0 (3)	C13—O6—C11—C12	−176.1 (4)
O7—C19—O8—Sm1^i^	−103.3 (7)	O4—C10—C11—O6	41.7 (6)
C20—C19—O8—Sm1^i^	81.0 (7)	O5—C10—C11—O6	−140.3 (4)
O8^i^—Sm1—N1—C35	−162.4 (3)	O4—C10—C11—C12	−75.8 (5)
O7—Sm1—N1—C35	109.3 (3)	O5—C10—C11—C12	102.3 (5)
O4—Sm1—N1—C35	−97.1 (3)	C35—N1—C38—C39	−3.6 (6)
O1—Sm1—N1—C35	25.5 (3)	Sm1—N1—C38—C39	173.9 (3)
O2—Sm1—N1—C35	33.3 (4)	C35—N1—C38—C37	176.7 (4)
O5—Sm1—N1—C35	−50.0 (3)	Sm1—N1—C38—C37	−5.8 (5)
N2—Sm1—N1—C35	−176.5 (4)	N2—C37—C38—N1	0.0 (5)
C1—Sm1—N1—C35	28.0 (3)	C36—C37—C38—N1	−178.2 (4)
C10—Sm1—N1—C35	−73.1 (3)	N2—C37—C38—C39	−179.7 (4)
O8^i^—Sm1—N1—C38	20.3 (4)	C36—C37—C38—C39	2.1 (6)
O7—Sm1—N1—C38	−68.0 (3)	C20—O9—C22—C23	1.6 (6)
O4—Sm1—N1—C38	85.6 (3)	C20—O9—C22—C27	179.1 (4)
O1—Sm1—N1—C38	−151.8 (3)	N1—C38—C39—C33	2.8 (6)
O2—Sm1—N1—C38	−144.0 (3)	C37—C38—C39—C33	−177.5 (4)
O5—Sm1—N1—C38	132.7 (3)	N1—C38—C39—C32	−179.3 (4)
N2—Sm1—N1—C38	6.2 (3)	C37—C38—C39—C32	0.4 (6)
C1—Sm1—N1—C38	−149.3 (3)	C38—N1—C35—C34	1.4 (6)
C10—Sm1—N1—C38	109.6 (3)	Sm1—N1—C35—C34	−175.9 (3)
Sm1—O2—C1—O1	3.7 (4)	C37—N2—C28—C29	0.4 (6)
Sm1—O2—C1—C2	−178.7 (3)	Sm1—N2—C28—C29	176.5 (3)
Sm1—O1—C1—O2	−3.8 (4)	C11—O6—C13—C18	13.9 (7)
Sm1—O1—C1—C2	178.8 (3)	C11—O6—C13—C14	−167.1 (4)
O8^i^—Sm1—C1—O2	−3.2 (3)	C38—C39—C33—C34	0.4 (7)
O7—Sm1—C1—O2	85.6 (2)	C32—C39—C33—C34	−177.4 (5)
O4—Sm1—C1—O2	−105.5 (2)	C29—C30—C36—C37	1.1 (7)
O1—Sm1—C1—O2	176.3 (4)	C29—C30—C36—C31	−178.8 (5)
O5—Sm1—C1—O2	−85.7 (2)	N2—C37—C36—C30	−1.2 (6)
N2—Sm1—C1—O2	110.2 (3)	C38—C37—C36—C30	177.0 (4)
N1—Sm1—C1—O2	171.0 (2)	N2—C37—C36—C31	178.7 (4)
C10—Sm1—C1—O2	−96.0 (2)	C38—C37—C36—C31	−3.2 (6)
O8^i^—Sm1—C1—O1	−179.5 (2)	C36—C30—C29—C28	−0.3 (7)
O7—Sm1—C1—O1	−90.7 (2)	N2—C28—C29—C30	−0.5 (7)
O4—Sm1—C1—O1	78.2 (3)	C39—C33—C34—C35	−2.6 (7)
O2—Sm1—C1—O1	−176.3 (4)	N1—C35—C34—C33	1.7 (7)
O5—Sm1—C1—O1	98.0 (3)	C2—O3—C4—C9	7.5 (7)
N2—Sm1—C1—O1	−66.1 (4)	C2—O3—C4—C5	−172.4 (5)
N1—Sm1—C1—O1	−5.3 (3)	C27—C22—C23—C24	−2.0 (8)
C10—Sm1—C1—O1	87.7 (3)	O9—C22—C23—C24	175.4 (5)
C28—N2—C37—C36	0.5 (6)	C5—C4—C9—C8	−1.3 (8)
Sm1—N2—C37—C36	−175.9 (3)	O3—C4—C9—C8	178.8 (5)
C28—N2—C37—C38	−177.7 (4)	C14—C13—C18—C17	−2.5 (8)
Sm1—N2—C37—C38	6.0 (4)	O6—C13—C18—C17	176.5 (5)
C22—O9—C20—C21	−160.5 (4)	C33—C39—C32—C31	176.0 (5)
C22—O9—C20—C19	81.0 (4)	C38—C39—C32—C31	−1.8 (8)
O7—C19—C20—O9	32.5 (5)	C39—C32—C31—C36	0.7 (9)
O8—C19—C20—O9	−151.6 (3)	C30—C36—C31—C32	−178.3 (5)
O7—C19—C20—C21	−84.8 (5)	C37—C36—C31—C32	1.8 (8)
O8—C19—C20—C21	91.2 (4)	C23—C22—C27—C26	2.4 (8)
O8^i^—Sm1—O5—C10	92.3 (3)	O9—C22—C27—C26	−175.3 (4)
O7—Sm1—O5—C10	−179.0 (2)	C13—C18—C17—C16	1.9 (9)
O4—Sm1—O5—C10	2.9 (2)	C26—C25—C24—C23	1.4 (9)
O1—Sm1—O5—C10	−129.7 (3)	C22—C23—C24—C25	0.1 (9)
O2—Sm1—O5—C10	174.2 (3)	C9—C4—C5—C6	−0.2 (10)
N2—Sm1—O5—C10	13.7 (3)	O3—C4—C5—C6	179.6 (7)
N1—Sm1—O5—C10	−58.9 (3)	C18—C17—C16—C15	−0.6 (9)
C1—Sm1—O5—C10	−157.4 (3)	C4—C9—C8—C7	1.1 (9)
O8—C19—O7—Sm1	7.0 (7)	C18—C13—C14—C15	1.9 (9)
C20—C19—O7—Sm1	−177.4 (3)	O6—C13—C14—C15	−177.2 (5)
O8^i^—Sm1—O7—C19	21.0 (4)	C24—C25—C26—C27	−1.1 (9)
O4—Sm1—O7—C19	109.9 (4)	C22—C27—C26—C25	−0.8 (9)
O1—Sm1—O7—C19	−115.8 (4)	C13—C14—C15—C16	−0.6 (10)
O2—Sm1—O7—C19	−60.3 (4)	C17—C16—C15—C14	0.0 (10)
O5—Sm1—O7—C19	−66.9 (5)	C4—C5—C6—C7	2.0 (13)
N2—Sm1—O7—C19	102.9 (4)	C5—C6—C7—C8	−2.3 (13)
N1—Sm1—O7—C19	169.5 (4)	C9—C8—C7—C6	0.7 (12)
C1—Sm1—O7—C19	−88.1 (4)		

Symmetry codes: (i) −*x*+2, −*y*+2, −*z*+1.
